# Newlavirus, a Novel, Highly Prevalent, and Highly Diverse Protoparvovirus of Foxes (*Vulpes* spp.)

**DOI:** 10.3390/v13101969

**Published:** 2021-09-30

**Authors:** Marta Canuti, Émilie Bouchard, Bruce Rodrigues, Hugh G. Whitney, Marti Hopson, Cornelia Gilroy, Garry Stenson, Suzanne C. Dufour, Andrew S. Lang, Joost T. P. Verhoeven

**Affiliations:** 1 Department of Biology, Memorial, University of Newfoundland, 232 Elizabeth Ave., St. John’s, NL A1B 3X9, Canada; hughwhitneynl@gmail.com (H.G.W.); sdufour@mun.ca (S.C.D.); verhoevenjtp@googlemail.com (J.T.P.V.); 2 Department of Veterinary Microbiology, Western College of Veterinary Medicine, University of Saskatchewan, 52 Campus Drive, Saskatoon, SK S7N 5B4, Canada; emilie_bou@hotmail.com; 3 Research Group on Epidemiology of Zoonoses and Public Health (GREZOSP), Faculty of Veterinary Medicine, Université de Montréal, 3200 rue Sicotte, Saint-Hyacinthe, QC J2S 2M2, Canada; 4 Wildlife Division, Newfoundland and Labrador Department of Fisheries, Forestry, and Agriculture, PO Box 2007, Corner Brook, NL A2H 7S1, Canada; brucerodrigues@gov.nl.ca; 5 Atlantic Veterinary College, University of Prince Edward Island, 550 University Ave., Charlottetown, PE C1A 4P3, Canada; mhopson@upei.ca; 6 Department of Pathology and Microbiology, Atlantic Veterinary College, University of Prince Edward Island, 550 University Ave., Charlottetown, PE C1A 4P3, Canada; cgilroy@upei.ca; 7 Fisheries and Oceans Canada, Government of Canada, P.O. Box 5667, St. John’s, NL A1C 5X1, Canada; garry.stenson@dfo-mpo.gc.ca

**Keywords:** protoparvovirus, parvovirus, virus discovery, fox, carnivore

## Abstract

The genus *Protoparvovirus* (family *Parvoviridae*) includes several viruses of carnivores. We describe a novel fox protoparvovirus, which we named Newlavirus as it was discovered in samples from Newfoundland and Labrador, Canada. Analysis of the full non-structural protein (NS1) sequence indicates that this virus is a previously uncharacterized species. Newlavirus showed high prevalence in foxes from both the mainland (Labrador, 54/137, 39.4%) and the island of Newfoundland (22/50, 44%) but was not detected in samples from other carnivores, including coyotes (*n* = 92), lynx (*n* = 58), martens (*n* = 146), mink (*n* = 47), ermines (*n* = 17), dogs (*n* = 48), and ringed (*n* = 4), harp (*n* = 6), bearded (*n* = 6), and harbor (*n* = 2) seals. Newlavirus was found at similar rates in stool and spleen (24/80, 30% vs. 59/152, 38.8%, *p* = 0.2) but at lower rates in lymph nodes (2/37, 5.4%, *p* < 0.01). Sequencing a fragment of approximately 750 nt of the capsid protein gene from 53 samples showed a high frequency of co-infection by more than one strain (33.9%), high genetic diversity with 13 genotypes with low sequence identities (70.5–87.8%), and no geographic segregation of strains. Given the high prevalence, high diversity, and the lack of identification in other species, foxes are likely the natural reservoir of Newlavirus, and further studies should investigate its distribution.

## 1. Introduction

Parvoviruses (family *Parvoviridae*, order *Piccovirales*) are small, non-enveloped single-stranded DNA viruses that infect both vertebrate and invertebrate animal species. The coding region of their genome, which is surrounded by imperfect palindromes that fold into terminal hairpins essential for DNA packaging and replication, includes two gene cassettes that encode non-structural (NS) and structural (VP) proteins. Gene expression is often regulated by a different promoter for each cassette and most transcripts are generated through alternative splicing [1].

The family *Parvoviridae* is divided into three subfamilies and the currently known viruses of carnivores (order Carnivora) belong to various genera within two of these subfamilies. These include the genus *Chaphamaparvovirus* of the subfamily *Hamaparvovirinae* and the genera *Amdoparvovirus*, *Bocaparvovirus*, *Dependoparvovirus*, and *Protoparvovirus* of the subfamily *Parvovirinae*. Viruses within the species *Carnivore chaphamaparvovirus 1* (Cachavirus), *Carnivore amdoparvovirus 2* and *3*, *Carnivore bocaparvovirus 1* and *2*, *Carnivore protoparvovirus 1, 3*, and *4* are known to infect canids (family Canidae) [2]. Parvoviruses known to infect foxes are mainly classified within the two genera *Amdoparvovirus*, which includes the Aleutian mink disease virus and several viruses recently identified in foxes and other carnivores [3,4,5,6], and *Protoparvovirus*, which includes important dog pathogens with broad host ranges and some additional recently discovered viruses. Among protoparvoviruses, *Carnivore protoparvovirus 1* (containing both canine parvovirus 2 (CPV-2) and feline panleukopenia virus (FPV)) [7], *Carnivore protoparvovirus 3* (canine Bufavirus (CBuV)) [8], and the *Carnivore protoparvovirus 4* (fox parvovirus) [9] have all been found in foxes. Still to be investigated is the host distribution of the remaining recently identified protoparvoviruses of marine carnivores, such as the sea otter parvovirus (species *Carnivore protoparvovirus 2*) [10,11], the fur seal parvovirus [12] and the California sea lion parvovirus [13].

An interesting feature of parvoviruses of carnivores is their wide host spectrum. For example, CPV-2 infection has been found in members of most families of carnivores, such as Canidae, Felidae, Mustelidae, and Procyonidae, and it has even been detected in a non-carnivore host, a pangolin [7,11,14,15]. Amdoparvoviruses are also known to infect different host species and at least three of the five amdoparvoviruses known to infect foxes have also been found in other species, including lynx, racoon dogs, mink, skunks, and martens [4,6]. Finally, infections caused by the recently discovered CBuV and Cachavirus have been found in various species of wild canids (CBuV) [8] and in dogs as well as cats [16,17]. It is therefore important to investigate the host spectrum of parvoviruses of carnivores to fully comprehend their ecology and the molecular mechanisms that shape their evolution.

In this study, we report the full molecular characterization and phylogenetic analysis of a novel fox protoparvovirus, which we named Newlavirus since it was discovered in animals from the Canadian province Newfoundland and Labrador. Additionally, we describe the epidemiology of this virus in the province and report preliminary data on virus dispersal, diversity, and tissue distribution, while investigating its potential for cross-species transmission to other carnivores.

## 2. Materials and Methods

### 2.1. Sample Collections

This study involved 729 samples collected from 613 terrestrial and marine carnivores, including 187 foxes (157 red foxes (*Vulpes vulpes*), 1 Arctic fox (*Vulpes lagopus*), and 29 foxes of unspecified species), 92 coyotes (*Canis latrans*), 48 free-roaming domestic dogs (*Canis lupus familiaris*), 58 lynx (*Lynx canadensis subsolanus*), 146 martens (*Martes americana*), 47 mink (*Neovison vison*), 17 ermines (*Mustela erminea*), and 18 seals (4 ringed seals (*Pusa hispida*), 6 harp seals (*Pagophilus groenlandicus*), 6 bearded seals (*Erignathus barbatus*), and 2 harbor seals (*Phoca vitulina*)). Samples included the spleen of 53 coyotes, 80 foxes, all lynx, martens, mink and ermines, paired rectal and tracheal swabs (polyester swabs, Starplex Scientific, Etobicoke, ON, Canada) submerged together in 3 mL universal transport medium (UTM, Starswab Multitrans System, Starplex Scientific, Etobicoke, ON, Canada) collected from seals, 6 rectal swabs in 3 mL UTM from 40 coyotes, rectal swabs in 400 µL UTM from healthy dogs, stool samples of 8 foxes, head lymph nodes of 27 foxes, paired stool and spleen for 62 foxes, paired rectal swabs and spleen for 34 coyotes, and matched head lymph node, stool and spleen for 10 foxes. A summary of tested samples is available in Table 1.

All samples were previously collected for other studies [6,18,19,20,21,22]. Samples from wild animals were obtained either from licensed trappers and hunters or wildlife regional offices, and animals were either killed for commercial purposes or found dead. Samples from dogs were collected during routine veterinary health examinations performed in association with the Chinook Project network [21].

### 2.2. Study Locations

**Figure 1:**
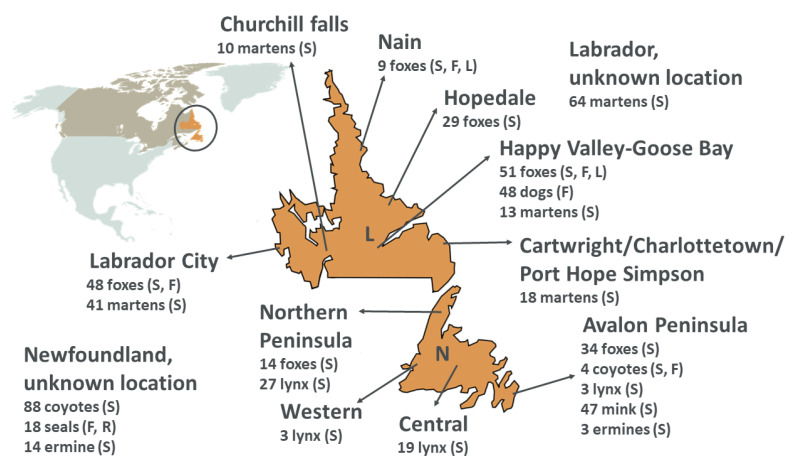
Locations of sample collection. The map on the top-left shows the province of Newfoundland and Labrador (included in the circle) within North America and Canada (brown), while Labrador (L) and the island of Newfoundland (N) are showed enlarged in the middle. For each location, the list of species and the number of investigated animals for each species are given together with the type of available samples (S: spleen; F: fecal; L: lymph node; R: respiratory). Map was created with Mapchart.net ^©^ (accessed on 28 September 2021).

The geographic area under investigation is Newfoundland and Labrador (NL), the easternmost province of Canada and the largest among the Atlantic provinces (Figure 1). Newfoundland is a large island of the North Atlantic Ocean located in the Gulf of St. Lawrence, while Labrador is a continental region bordering Quebec. The two portions of land are separated by at least 18 km of water at the closest point, but terrestrial animals occasionally cross via connecting ice bridges and may occasionally move from the mainland to Newfoundland on icebergs or ice floes.

Foxes (*n* = 50), lynx, mink, ermines, and coyotes were sampled in Newfoundland, while fox (*n* = 137), marten, and dog samples originated from Labrador. Seals were collected in Newfoundland but are known to frequent both Newfoundland and Labrador waters. Sampling locations included four different regions of Newfoundland, and areas surrounding several communities in Labrador (Figure 1). 

### 2.3. Molecular Methods

Virus discovery was initially performed on 68 stool samples collected from Labrador foxes with the VidION method as previously described [23]. Before sample pre-treatment, an approximately 0.5 cm^3^ piece of stool was placed in a 2-mL screw-capped tube with 1 mL UTM and three 4.5-mm copper-coated steel beads (Crosman, Walmart, Mississauga, ON, Canada) for vortex-mediated fragmentation. After mixing, the tubes were briefly spun down at low speed and 210 µL of supernatant were used as input for VidION.

For complete genomic sequencing, screening, and molecular epidemiology assays, DNA obtained during previous studies [11,18,19,20] from 10 mg spleen, 25 mg lymph node, or from the samples used for virus discovery was used. Additionally, DNA isolated with the DNeasy Blood and Tissue Kit (Qiagen, Toronto, ON, Canada) from 90 µL of seal respiratory and fecal material resuspended in UTM, or 200 µL fecal suspension from dogs and coyotes were used for screening.

Genomic sequencing was performed with the ViDiWa genome walking method [24] and screening was performed with two heminested PCRs with specifically designed primers. Initially, all fox samples were screened with primers SlPV_F1 (5′-GTGCTAAGAGGCTTCTTGCG-3′) and SlPV_R1 (5′-GTACGATTGTCAAAATTGCCAG-3′) during the first PCR and SlPV_F2 (5′-ACTCCTCTACCGAAACATCC-3′) and SlPV_R1 during the nested-PCR, amplifying a 355-nt fragment of the VP1 gene. After discovering higher-than-expected sequence variation, new primers were designed and all samples that remained negative were screened a second time with primers SlPV_ScF (5′- AGACACTGACCAAGCACCC-3′) and SlPV_R1 during the first PCR and SlPV_ScF and SlPV_ScR (5′-CTCAGGTTCCATTGGCTCG-3′) for the nested PCR, amplifying a 328-nt portion of the VP1 gene. All other samples were screened with the second primer set. To study the molecular epidemiology of the identified strains, a 736–745-nt fragment of the VP1 gene was amplified from positive fox samples with primers SlPV_F3 (5′-CTGGCAATTTTGACAATCGTACG-3′) and SlPV_R10 (5′-GGCGGTGCACCAAGCATTC-3′) during the first PCR and SlPV_F12 (5′-TTGACAATCGTACGCTTTGGC-3′) and SlPV_R10 for the nested PCR. All PCRs were performed with the DreamTaq Green PCR Master Mix (Thermo Fisher Scientific, Waltham, MA, USA) and positive samples were purified with AMPure XP beads (Beckman Coulter, Brea, CA, USA) and outsourced for Sanger sequencing.

### 2.4. Bioinformatic Methods and Statistical Analysis

Sequence quality checking, assembly, open reading frame (ORF) predictions, and annotations were performed in Geneious R11 (Biomatters, Auckland, New Zealand). Additionally, splicing donor and acceptor sites were predicted with NNSPLICE 0.9 [25] and promoter regions were predicted with NNPP 2.2 [26]. Similarity plots were obtained with Simplot 3.5 [27].

Alignments were made with MAFFT [28] with the E-INS-I algorithm and maximum likelihood phylogenetic trees were built with IQ-TREE 2 [29] using the best fit substitution model identified as the one with the lowest Bayesian information criterion (BIC) with the ModelFinder function [30]. Branch support was assessed with both ultrafast bootstrap approximation (ufBoot) [31] and SH-like approximate likelihood ratio test (SH-aLRT) [32]. A dataset including representatives of all known protoparvoviral species and two fox amdoparvoviruses as an outgroup was used for virus classification. Trees were annotated in MEGA X [33] and final figures prepared with INKSCAPE (https://inkscape.org/, downloaded on 19 June 2020).

Differences in positivity rates (number of positive samples over the total number of samples) between groups were evaluated for statistical significance using the Mid-p exact test with OpenEpi [34], and *p*-values ≤ 0.05 (two-tailed tests) were considered significant.

## 3. Results

### 3.1. Genome Characterization

**Figure 2:**
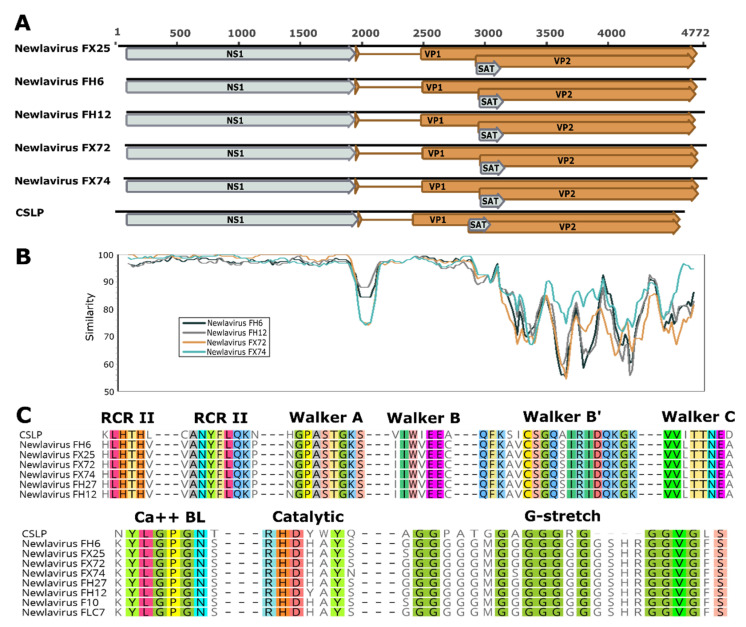
Genomic features of five Newlavirus strains compared to those of the California sea lion parvovirus (CSLP). (**A**): schematic genome representations with in silico predicted ORFs. (**B**): nucleotide identities throughout the genomes of four strains compared to the FX25 reference strain. (**C**): conserved motifs typical of parvoviruses. Rolling circle replication (RCR) and Walker motifs typical of NS1 are shown at the top and phospholipase A2 (Ca++ BL: calcium binding loop; Catalytic: catalytic site) and glycine-rich (G-stretch) motifs typical of VP1 at the bottom.

After discovering the first Newlavirus fragment with the VidION method [23], the near complete genomes of 5 strains were obtained through a combination of genome walking [24] and specific PCRs designed based on newly obtained sequences. The sequences encompass the entire coding portion but lack the inverted non-coding repeats that form the terminal hairpins, which are difficult to obtain from non-cultured viruses (Figure 2). Genomes were 90.4–94% identical to each other.

All viruses presented the typical parvoviral genomic structure with the two coding cassettes for the NS1 and VP1/VP2 proteins. A promoter could be identified in the 3′ terminal part of the NS1 region of all viruses, suggesting that the structural and non-structural genes are transcribed from two different promoters. Comparing the Newlavirus genomes with their closest known relative, the California sea lion parvovirus (CSLP) [13] (see below), revealed a conserved pattern with two big ORFs coding for the NS1 (617 amino acids) and VP2 (586–590 amino acids) proteins. Additionally, a short ORF was present in all viruses starting 7 nucleotides downstream of the start of VP2 (Figure 2). This showed homology to the small alternatively translated (SAT) protein (63–64 amino acids) of the *Rodent protoparvovirus 1* and *Carnivore protoparvovirus 1*.

Additionally, we could observe for all viruses a splicing donor site just downstream of the NS1 stop codon and an acceptor site 503–515 nt afterwards that could be connected to generate the ORF for the VP1 protein (751–755 amino acids). A similar structure, but with a slightly shorter gap (417 nt), was observed for the CSLP. Although several splicing donor sites conserved across strains could be observed at the beginning of NS1, we could not identify any donor/acceptor sites that could give rise to ORFs of other potential non-structural proteins. However, the high sequence conservation in the area upstream of the second exon of VP1 could suggest the existence of a NS exon in this area.

Viruses showed high sequence similarities over the whole lengths of NS1 and the VP1 unique region, while sequence identities dropped significantly in the VP2 area. In fact, nucleotide sequence identities in the NS1 region were 97.2–98.8% compared to identities of 79.3–90.2% for the VP2 gene. In the conserved parts of the genome the typical protein motifs of parvoviruses could be identified. These included the rolling circle replication motifs II and III and the four Walker domains typical of the helicase NS1, and the phospholipase A_2_ domain and the glycine-rich stretch typical of parvoviral capsid proteins [1] (Figure 2).

### 3.2. Viral Classification

Parvovirus classification is based on NS1 protein sequence phylogeny, as this contains the most conserved and family-characterizing helicase superfamily 3 (SF3) domain [2]. According to the parvovirus classification system, viruses that diverge more than 15% in amino acid sequence identity are considered different species and groups of viruses that form a bootstrap-supported clade and whose sequence identity is at least 30–40% are considered one genus [2].

**Figure 3:**
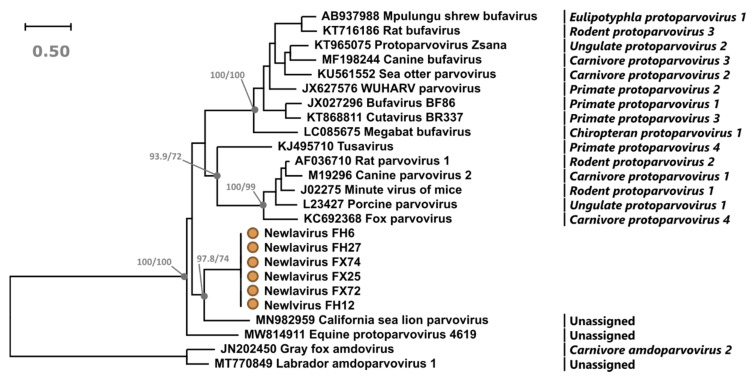
Phylogenetic analysis of Newlavirus and other protoparvoviruses. The phylogenetic tree based on the full NS1 protein sequences was built with the maximum-likelihood method with the rtREV + F + I + G4 model using IQ-Tree. The outcomes of the SH-aLRT and bootstrap test (1000 replicates) are shown for the main nodes. The viruses identified in this study are labelled with an orange circle and species designations, when available, are indicated on the right.

According to this definition, all sequenced Newlavirus strains, although very divergent from each other in the VP2 region, constitute the same viral species as their NS1 amino acid sequences were 98.6–99.5% identical. Furthermore, phylogenetic analyses clearly showed that Newlavirus belongs to the genus *Protoparvovirus*, as its full NS1 amino acid sequences formed a bootstrap-supported clade with representatives of all known [2] and recently discovered [13,35] protoparvovirus species (Figure 3). Interestingly, in this tree, three distinct and well supported groups of protoparvoviruses could be observed with two of them including all of the already classified viruses.

NS1 proteins of Newlavirus strains were 38.7–54.1% identical to members of the genus *Protoparvovirus* and their closest relatives among the strains used for the phylogenetic analysis were the recently discovered and not yet officially classified CSLP (53.6–54.1%) and equine protoparvovirus (46.1–46.7%). According to a blast analysis, however, the closest relative of Newlavirus was the fur seal parvovirus (62.8–63.4% partial NS1 amino acid identity) [12], which is the closest known relative of CSLP (74.9%) but whose genome was only partially sequenced and it was therefore not included in the phylogenetic analysis.

### 3.3. Molecular Epidemiology and Distribution

To evaluate Newlavirus host distribution, we screened 729 samples collected from 613 carnivores (Table 1) and none of the samples collected from coyotes, dogs, lynx, martens, mink, ermines, or seals tested positive. However, a high viral prevalence was found in foxes (40.6%) and the virus was detected in both red foxes and in the one sample available from an Arctic fox (Table 2).

Interestingly, the virus was present at significantly higher rates in spleen and stool compared to lymph nodes (*p* < 0.001 and *p* < 0.002, respectively) while positivity rates were comparable for spleens and stool. Prevalence rates were comparable in all studied locations with the exception of the area of Happy Valley-Goose Bay, which showed a lower rate. However, for about half of the animals from this area (*n* = 25) the only available sample was a lymph node and the low detection rate here could reflect a sample-type bias. This was also confirmed by the fact that no lymph node samples from Nain, where viral prevalence was 55.6%, were positive. Among positive animals for which both spleen and stool were available, we observed a concordance of 27.3% (9/33).

All fox samples were previously screened for amdoparvoviruses [6] and, while amdoparvoviral prevalence in this population was 3.2%, only one animal presented with a co-infection with Aleutian mink disease virus.

**Figure 4:**
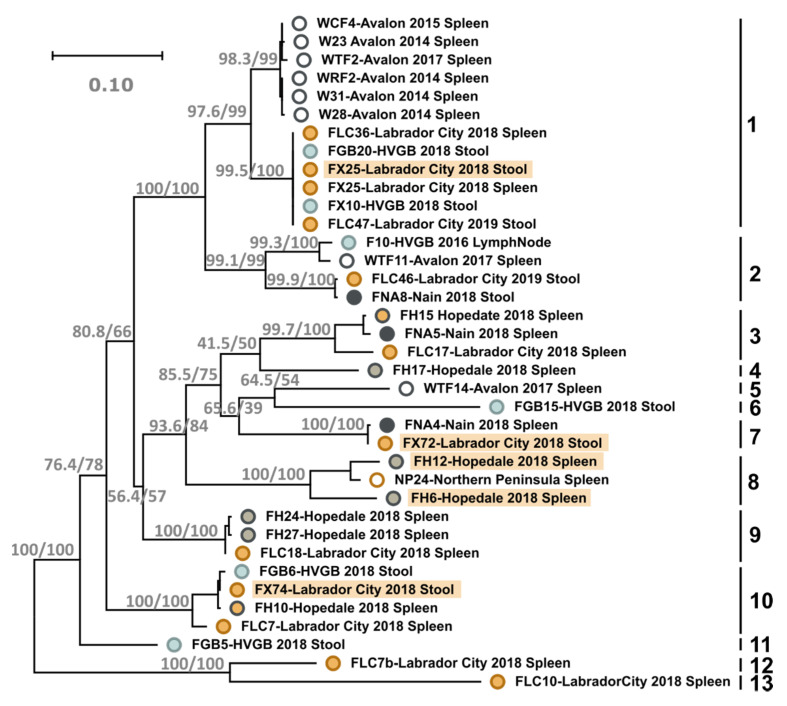
Phylogenetic analysis of Newlavirus strains identified in this study. The unrooted phylogenetic tree is based on partial VP1 nucleotide sequences and was built with the maximum-likelihood method based on the GTR + F+I + G4 model with IQ-Tree. The outcomes of the SH-aLRT and bootstrap test (1000 replicates) are shown for the main nodes. The viruses identified in this study are labelled with colored circles corresponding to the sampling locations. Each sequence name includes the viral strain identifier, sampling collection site and type of sample in which the virus was identified. The 13 viral genotypes are indicated on the right and strains whose complete genomes were sequenced are highlighted in orange. HVGB: Happy Valley-Goose Bay.

For 53 of the positive animals, we were able to amplify and sequence a fragment of approximately 740 nt of the VP1 gene and 18 of those (33.9%) showed the presence of more than one strain, indicated by multiple occurrences of double peaks in the electropherograms, demonstrating a high co-infection rate. The remaining sequences (70.5–100% identical to each other) could be subdivided into 13 different phylogenetically supported genotypes, which we defined as groups characterized by a within pairwise sequence identify of >89% or single-virus clades diverging >10% from all other viruses (Figure 4).

Since most of the obtained sequences were from Labrador, most clades included only sequences from this region and sequences from Newfoundland were observed in only 4 clades. While no clear geographic segregation of viruses was observed, as each multi-sequence clade contained viruses from various geographic locations, evidence for local differentiation could be observed in clade 1, which was the clade with most sequences, where sequences from Newfoundland and Labrador formed two distinct sub-clades.

## 4. Discussion

The mammalian order Carnivora is characterized by a large diversity of members, which include both terrestrial and aquatic species that naturally occur in all continents and oceans [36]. Because of their feeding behavior, which involves hunting and carcass scavenging, these animals are constantly exposed to a high number of infectious agents and play a crucial role in disease spread, especially considering that many carnivore parasites are multi-host, i.e., capable of infecting multiple species [37,38]. Identifying microorganisms of carnivores and studying their host distribution and transmission dynamics has, therefore, important implications for animal health and conservation programs [39], especially since cross-species transmission does happen frequently and sometimes with catastrophic consequences [37,40]. In this manuscript, we fully characterized and investigated the epidemiology and host distribution of Newlavirus, a novel parvovirus we discovered in foxes.

### 4.1. Host-Specificity of Newlavirus Compared to Other Carnivore Parvoviruses

The genus *Protoparvovirus* currently includes 17 species, 15 official and two proposed [1,2,13,35], five of which are viruses of carnivores including three detected at least once in foxes [8,9,15]. Newlavirus, which fulfills all the requirements established by the ICTV to be classified as a parvoviral species is, to the best of our knowledge, now the sixth protoparvovirus of carnivores to be fully sequenced.

Among all known species, the best studied protoparvovirus is likely the *Carnivore protoparvovirus 1*, a species that includes CPV-2, a virus that is known for infecting a broad range of hosts in the order Carnivora [7,11,15] and that was even found in a non-carnivore host [14]. Additionally, CBuV (*Carnivore protoparvovirus 3*), which is the only other protoparvovirus of carnivores for which epidemiological investigations have been published, has been found in several different species, including both felids and canids [8,17,41]. Similarly, the genus *Amdoparvovirus*, whose members are the closest known relatives to protoparvoviruses [2,11,42], includes several viruses that have the capability of infecting different carnivore hosts when the chance for cross-species transmission is presented [6].

Differently from these well-known examples, Newlavirus shows specificity for foxes since none of the 427 samples collected from other carnivores, including two other canine species, were found to be positive. Although the number of tested carnivore species was low and does not represent an exhaustive list, we would expect to identify some positive animals if this virus frequently crossed the species barrier. With a prevalence of 40% in its maintenance host and the fact that the virus is frequently found in tissues and stool, the chance for spill-over is high, especially considering the high environmental resistance of parvoviruses [1] and the fact that carnivorous and scavenging lifestyles facilitate pathogen exchange among carnivores [6,11]. Additionally, in studies that examined the same samples used for this investigation for other parvoviruses [6,21], we documented that cross-species transmission of parvoviruses occurs frequently in carnivore populations of Newfoundland and Labrador. However, in a multi-host and multi-pathogen system, virus exchange is not always as frequent as it might be predicted on the basis of habitat overlap, and cross-species transmission may not be multidirectional (e.g., a virus might be more easily transferred from prey to predator as opposed to the other way around) [11]. Viral distribution may therefore vary among different hosts, and it is possible that cross-species transmission of Newlavirus does occur but not frequently enough for us to detect it. Further studies investigating a larger number of samples from a wider species range are essential to elucidate this aspect.

### 4.2. Newlavirus in Foxes

Newlavirus was discovered in a stool sample, but subsequent investigations showed that the virus is also detectable in tissue samples, indicating that it is not a food-borne contaminant and that it causes an active and systemic infection in foxes. However, the high prevalence (40.6%) could suggest low pathogenicity. Interestingly, while positivity rates for stool samples and spleens were comparable (30.0% vs. 38.8%), Newlavirus was present at significantly lower rates in lymph nodes collected from the head (5.4%).

Our results might suggest that Newlavirus possesses enteric tropism, similar to its close relative CPV-2, which causes severe diarrhea with associated lymphopenia consequent to damage of the cells of the gastrointestinal tract and lymphoid tissues [43,44]. This is corroborated by the fact that parvoviruses require cells with high mitotic index to replicate and epithelial and lymphoid tissues are often targets of viral replication [1,45,46]. A higher positivity rate in spleen versus lymph nodes might also suggest viral accumulation in the spleen following past infections, while positive lymph nodes might be indicative of a current infection associated with viremia. Unfortunately, the only two lymph nodes that tested positive in our study were collected from animals for which only these tissues were available. The six animals that were positive in other sample types and for which lymph nodes were also available showed a variable pattern of positivity (two of them presented virus in both spleen and stool, two only in spleen, and two only in stool). Finally, a higher detection rate in feces compared to lymph nodes might indicate either that the virus has higher affinity for intestinal epithelial cells or that shedding is persistent. This is also consistent with the low percentage (<30%) of infected animals that tested positive for the virus in both spleen and stool. An alternative hypothesis could be viral accumulation in spleens and vascular tissues with consequent viral shedding in feces, similar to what is observed for the Aleutian mink disease virus [47]. Since no data about the health status of the sampled animals were available, these hypotheses will have to be evaluated by clinical studies and pathological examinations. Specifically, in situ hybridization methods will help understanding tissue distribution and viral culture on cell lines could clarify viral replication strategies, including which receptors are required for viral entry.

Newlavirus was highly prevalent in all investigated fox populations, which were located across considerable distances both in mainland Canada (Labrador) and on the island of Newfoundland. In fact, the linear distance between the two furthest away sampling locations was over 1000 km and positive animals were also detected in remote areas of northern Labrador. We can confidently conclude that the virus is widely diffused among foxes of Atlantic Canada and probably also beyond this region. Although only one of the samples included in this study was collected from an Arctic fox, we found evidence for infection in two fox species within the genus *Vulpes* (*V. vulpes* and *V. lagopus*) and it is possible that this virus is capable of infecting other related species. Future studies should, therefore, investigate viral presence in different species of foxes from various locations to clarify this aspect. It would be interesting to elucidate, for example, if Newlavirus is widespread in red and Arctic foxes of North America, Asia, and Europe and if the virus can infect other fox species of North America, such as the grey fox (*Urocyon cinereoargenteus*) or the kit fox (*Vulpes velox*).

### 4.3. Newlavirus Diversity

A very high genetic variability was identified among the sequences with 13 viral strains differing considerably in their capsid protein gene sequences (with a nucleotide identity between strains as low as 70%). Interestingly, all sequenced NS genes showed high identity, confirming that all viruses belong to the same viral species. However, we did not manage to obtain the complete genomic sequences of some of the most divergent types (e.g., strain FL10) and these might represent additional novel species. The high variability of the capsid indicates that this region is likely under positive selection pressure due to the host immune response and that the virus has circulated among foxes for a long time and had the time to diverge into so many different types.

When we looked at the distribution of the different genotypes, we observed that samples collected from distant locations, including the island and the mainland, clustered together and their partial VP sequences were sometimes almost identical. This indicates a past, and likely current, viral dispersal across long distances. However, viruses from Newfoundland were never identical to those from Labrador and, within type 1, which included the highest number of sequences, viruses from the two different areas formed two separate clades. This indicates that, although viral exchange between the two portions of land can occur and it is probably made possible by the formation of ice bridges that animals can cross, viruses do establish efficient local transmission and can give rise to regional variants. This complicated evolutionary history also points towards a long-term association between the virus and its host.

## 5. Conclusions

In this study we describe the characterization and molecular epidemiology of a novel protoparvovirus we discovered in foxes in Newfoundland and Labrador. Although several aspects of viral ecology were investigated, future studies will have to confirm our findings and assess whether the virus is indeed restricted to foxes and define its geographical distribution. The discovery of this highly prevalent and highly variable virus increases our knowledge and understanding of parvoviruses of carnivores that, although genetically related to each other, vary considerably in terms of ecology, distribution, epidemiology, and evolutionary dynamics. Future virus discovery studies will be pivotal to identify additional yet unknown viruses and explore in depth the ecology and pathogenicity of different parvoviruses, which seem to be so amazingly diverse and widespread among carnivores.

## Figures and Tables

**Table 1:** Samples included in this study.

Animal	N. Animals	N. Fecal Samples	N. Tissues
Fox	187	80	179
Coyote	92	40	87
Dog	48	48	0
Lynx	58	0	58
Mink	47	0	47
Marten	146	0	146
Ermine	17	0	17
Ringed seal	4	4	0
Harp seal	6	6	0
Bearded seal	6	6	0
Harbor seal	2	2	0

**Table 2:** Detection rates of Newlavirus in foxes.

	N. Tested ^1^	N. Positives	% Positive
Red fox	157	60	38.2
Arctic fox	1	1	100
Unknown fox species	29	15	51.7
Total	187	76	40.6
Newfoundland	50	22	44.0
Labrador	137	54	39.4
Avalon Peninsula	34	14	41.2
Northern Peninsula	14	7	50.0
Nain	9	5	55.6
Hopedale	29	15	51.7
Labrador City	48	24	50.0
Happy Valley-Goose Bay	51	10	19.6
Lymph nodes	37	2	5.4
Spleen	152	59	38.8
Stool	80	24	30.0

^1^ Number of animals (species and location) or number of samples (sample type).

## Data Availability

Sequences obtained in this study have been deposited in GenBank under accession numbers MZ813278–MZ813314.
